# Efficacy and safety of percutaneous cement discoplasty in the management of degenerative spinal diseases: A systematic review and meta-analysis

**DOI:** 10.1177/19714009231212368

**Published:** 2023-11-03

**Authors:** Sahibjot Singh Grewal, Joshua A Hirsch, Nicole M Cancelliere, Sherief Ghozy, Vitor Mendes Pereira, Adam A Dmytriw

**Affiliations:** 1 Neurovascular Centre, Departments of Medical Imaging & Neurosurgery, St. Michael's Hospital, 7938 University of Toronto, Toronto, ON, Canada; 2 Neuroendovascular Program, 2348 Massachusetts General Hospital & Brigham and Women's Hospital, Harvard Medical School, Boston, MA, USA; 3 198511 Department of Radiology, Mayo Clinic, Rochester, MN, USA; 4 Nuffield Department of Primary Care Health Sciences and Department for Continuing Education (EBHC program), Oxford University, Oxford, UK

**Keywords:** Degenerative, spine, discoplasty, interventional neuroradiology

## Abstract

**Background:**

Percutaneous cement discoplasty (PCD) is a minimally invasive procedure. We aim to explore the efficacy and indication(s) of PCD in patients with degenerative disc disease (DDD).

**Methods:**

The search was conducted across Ovid MEDLINE, Ovid Embase, and PubMed. Data on study design, patient demographics, pre- and post-procedure Visual Analog Scale (VAS) and Oswestry Disability Index (ODI) scores, and complications were extracted. Inclusion criteria focused on adult patients with degenerative spinal diseases treated with cement discoplasty. The overall effect size was evaluated using a forest plot, and heterogeneity was assessed using the I^2^ statistic and chi-squared test.

**Results:**

The search strategy yielded six studies, which included 336 patients (73.8% female, 26.2% male) with a mean average age of 74.6 years. VAS scores were reported in all studies, showing a significant difference between pre- and post-PCD pain scores (Weighted Mean Difference [WMD]: -3.45; 95% CI: -3.83, -3.08; I^2^ = 15%; P < .001). ODI scores were reported in 83% of studies, with a significant difference between pre- and post-PCD scores (WMD: -22.22; 95% CI: -25.54, -18.89; I^2^ = 61%; *p* < .001). Complications reported included infections, thrombophlebitis, vertebral fractures, disc extrusion, and the need for further operations.

**Conclusions:**

The analysis showed clinically significant improvements in pain and functional disability based on VAS and ODI scores. However, due to methodological limitations and a high risk of bias, the validity and generalizability of the findings are uncertain. Despite these issues, the results provide preliminary insights into PCD's potential efficacy and can guide future research to address current limitations.

## Introduction

Degenerative disc diseases (DDD) are a common source of chronic low back pain, affecting 266 million people worldwide.^1^ The condition is characterized by the gradual breakdown of the intervertebral discs in the spine, leading to changes in disc morphology that make the spine less stable.^2^

Physical therapy is often the first line of treatment for DDD, and it typically involves exercises and stretches to improve flexibility and strength, as well as pain management techniques.^2,3^ While physical therapy can be effective for some patients, it may not be sufficient to alleviate the pain and other symptoms of DDD. Medications such as non-steroidal anti-inflammatory drugs (NSAIDs), opioids, and muscle relaxants may be used to manage the pain associated with DDD.^2,3^ However, these medications are not effective for long-term pain management and have no effect on the progression of disc degeneration.^3^

Surgery is generally reserved for patients who have not responded to non-surgical treatments or who have severe or progressive symptoms.^3^ Surgical options for DDD include decompression, artificial disc replacement, and spinal fusion.^3^ While surgery can effectively reduce pain and improve mobility, it is also associated with significant risks and complications, and it may not be suitable for all patients.^3^

Despite these treatment options, DDD remains a significant burden on patients and healthcare systems, and therefore there is a need for alternative treatment options.^1^ One such option is percutaneous cement discoplasty (PCD), a minimally invasive procedure that offers a potential remedy for individuals suffering from degenerated intervertebral discs.^4^ This technique involves the injection of poly-methylmethacrylate (PMMA) directly into the affected disc, precisely guided by medical imaging, as originally introduced by Varga et al.^4^ PCD stabilizes degenerated discs, characterized by the vacuum phenomenon, and provides indirect foraminal decompression. By achieving these objectives, the procedure aims to alleviate pain and enhance mobility in patients.^4,5^

This manuscript intends to examine the existing literature on the utilization of PCD in treating DDD. While Fusini et al.^6^ conducted a systematic review exploring the indications, clinical outcomes, and complications associated with PCD in DDD, no meta-analysis has been published on this topic. Considering the limited number of studies published on this technique,^7^ our objective is to comprehensively review the literature to encompass any recent publications, establish an effect size of the intervention, and evaluate the consistency of results across studies, offering insights into the generalizability of the findings.

## Materials and methods

The current study was conducted following the Preferred Reporting Items for Systematic Review and Meta-Analysis (PRISMA) checklist. Study Protocol was proactively registered at its onset in the University of York—PROSPERO database (ID: CRD42023405770).

### Search strategy and study selection

Pre-defined search terms were then used to search three databases. Included databases were Ovid MEDLINE®, Ovid Embase®, and PubMed. These searches were done with the following mesh terms: ((cement AND discoplast*) AND ((verteb*) OR (spin*) OR (degenerat*))). The search results were uploaded onto Covidence, and duplicate studies were removed through this platform.

Two reviewers assessed the eligibility criteria for the inclusion of studies, both at the title and abstract stage and the full-text stage. Each reviewer independently evaluated the studies for inclusion and was blinded to each other's decisions. The results of these assessments were documented on Covidence. Inclusion criteria followed the PICO format, where the population was adult patients (>18 years) who had been diagnosed with a degenerative spinal disease, including conditions such as spinal osteoarthritis, degenerative spondylolisthesis, and degenerative scoliosis. The intervention was cement discoplasty procedure performed for the management of degenerative spinal disease. No control or comparison was defined, and all efficacy outcomes were included. Case series, case–control studies, comparative studies, and randomized/quasi-randomized control trials that had been published from 2010 onwards were all included. Exclusion criteria were pediatric patients (<18 years), cement discoplasty procedures performed for the management of other conditions or diseases, unpublished studies, studies where patients received treatment other than PCD, systematic reviews, conference proceedings, articles before 2010, articles not in English, and grey literature.

### Data extraction

For data extraction, two review authors independently screened titles and abstracts of articles that may be eligible based on the inclusion criteria. Subsequently, the full text of these eligible articles was retrieved and screened to determine if they met the inclusion criteria. Any disagreements were addressed through consensus. Data extraction included information on the study design and methods, patient demographics, pre-procedure and post-procedure Visual Analog Scale (VAS) and Oswestry Disability Index (ODI) scores, and complications from the procedure.

### Quality assessment

The overall review adhered to the Preferred Reporting Items for Systematic Reviews and Meta-Analyses (PRISMA) standards.^8^ The quality of each study was assessed individually. The internal validity of each study was analyzed using the Methodological Index for Non-Randomized Studies (MINORS) tool.^9^

### Data analysis

The VAS and ODI indicators were used to make forest plots and evaluate the results. For studies where the post-procedure indicators were reported at various time points, the value closest to a 6-month follow-up was included in the analysis for consistency.

A within-group meta-analysis was performed on RevMan 5.4 software.^10^ For each study, the mean difference was calculated using the pre-intervention and post-intervention scores. These continuous outcomes were expressed in terms of mean difference and 95% CI. The mean differences were then pooled across studies using random-effects model, and the overall effect size was evaluated using a forest plot. Random-effects model was used due to methodological heterogeneity among the included studies. Heterogeneity was assessed among the studies using the I^2^ statistic and the chi-squared test. Heterogeneity was interpreted as low (I^2^ < 30%), moderate (30% ≤ I^2^ < 60%), or high (I^2^ ≥ 60%), with a chi-squared test *p*-value ≤ .05 considered statistically significant.

### Risk of bias

The Risk of Bias in Non-Randomized Studies of Interventions (ROBINS-I) tool was used to assess the risk of bias for included studies in this paper.^11^

## Results

### Search results

**Figure 1.**
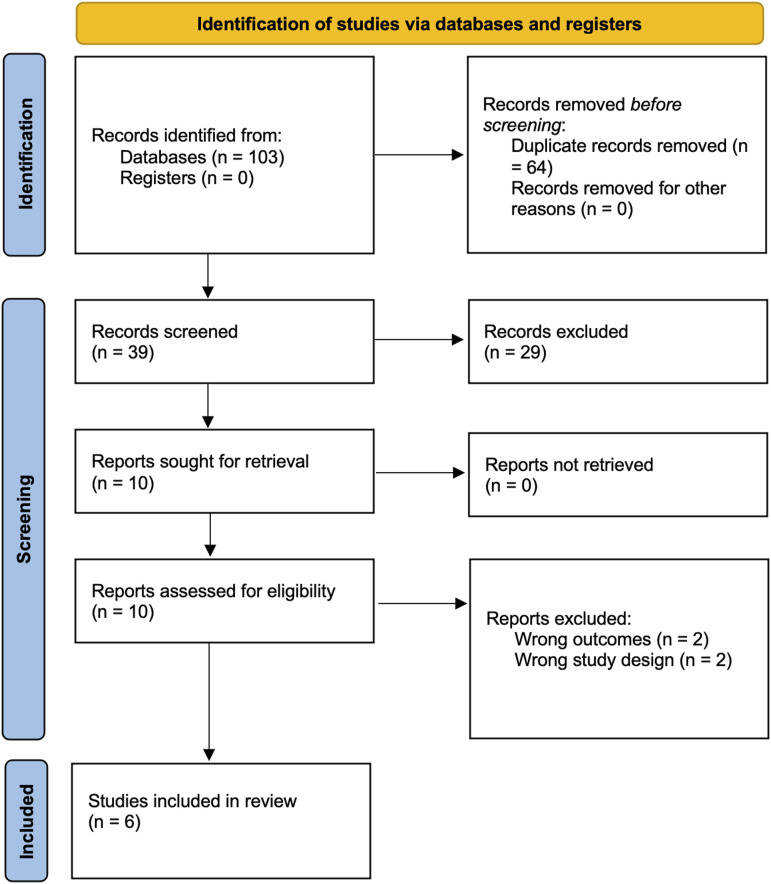
Preferred Reported Item for Systematic review and Meta-Analysis of the articles retrieved from the databases search included in the SR-MA(8).

The search strategy yielded a total of 103 studies after an integrated search across the three databases. After removing duplicates, there was a total of 39 studies selected for the title and abstract screening. This screening yielded ten full-text studies for further screening, and finally, six studies were included in the review based on the pre-determined criteria. Data were then extracted from these papers. The systematic search is summarized in a PRISMA flowchart in Figure 1.

### Study characteristics

Through six full-text articles, a total of 336 patients were collected. Of these, 248 patients were female (73.8%), and 88 (26.2%) were male, with a mean average age of 74.6 years. Degenerative disc disease was the diagnosis for 170 (50.6%) patients, degenerative scoliosis was the diagnosis for 106(31.5%) patients, degenerative spondylolisthesis was the diagnosis of 15(4.5%) patients, adjacent segment disease was the diagnosis for 15(4.5%) patients, lumbar degeneration with lumbar stenosis was the diagnosis for 14(4.2%) patients, combined pathologies was the diagnosis for 12(3.6%) patients, and non-union was the diagnosis for 5(1.49%) patients (Table 1).

**Table 1.** Summary of the included studies.

Study ID	Total number of participants	Gender	Age (yrs)	Spinal pathology	Pre-procedure VAS score (LBP)	Post-procedure VAS score (LBP)	Pre-procedure ODI score	Post-procedure ODI score	Complications from PCD
Michael et al., 2022	6	4F, 2M	74.3 (65–86)	Degenerative disc disease (*n* = 5), degenerative disc disease with spondylolisthesis (*n* = 1)	8.4+/- 0.57	2.8+/-3.1	Not reported	Not reported	1 case of fusion surgery due to persistent pain
Kiss et al. (2019)	28	7M, 21F	75.4 ± 7.4	Degenerative disc diseases (*n* = 28)	5.9+/-3.0	3.5+/-2.5	55.4 ± 13.9	37.9 ± 21.4	Not reported
Camino-Willhuber et al. (2021)	156	41M, 115F	75.8+/-5.7	Degenerative scoliosis (*n* = 67), advanced disc degeneration without scoliosis (*n* = 29), adjacent segment disease (*n* = 15), lumbar degeneration combined with lumbar stenosis (*n* = 14), spondylolisthesis (*n* = 12), combined pathologies (*n* = 12) Non-union (*n* = 5)	7.8 +/-0.9	3 months: 4.2 6 months: 3.70 12 months: 3.52 24 months: 3.56	68.1 +/- 9.6	12 months: 19.13 24 months: 17.18	15 patients required unplanned readmission within 30 days, which required intravenous medication
									5 cases of decompression surgery
									5 cases of superficial skin infections
									5 cases required PCD in another level due to pain recurrence
									4 cases required fusion surgery due to persistent pain
									3 cases of urinary tract infections
									2 cases of deep infections
									2 cases of superficial thrombophlebitis
									1 case of deep vein thrombosis
									1 case of adjacent vertebral fracture
Alkharsawi et al. (2022)	45	30F, 15M	74+- 6.5	Degenerative lumbar spine disease	7.5 (range 6–9)	3.5 ± 2.3 (range 2–5) (*p* = .02)	36 (range 30–45)	12.3 ± 4.8 (range 5–20) (*p* = .001)	2 cases of reoperation due to the symptom persistence
Camino-Willhuber et al. (2020)	54	43F, 11M	76 (69–87)	Degenerative scoliosis (*n* = 37), advanced degenerative disc disease without scoliosis (*n* = 15), degenerative spondylolisthesis (*n* = 2)	7.8 + -0.90	4.4 + -2	62 + 7.12	36.2 + 15.47	1 case had a vertebral fracture adjacent to the discoplasty (required a vertebroplasty)
									2 cases of foraminal decompression (due to radicular symptoms)
									2 cases of cement leakage to the foramen
									1 case of disc extrusion experienced by a patient
									1 case of deep infection
Varga et al. (2015)	47	35F, 12M	69.2 (44–84)	Degenerative disc diseases	Mean of 6.8	Mean of 4.3	50.34 (range 12–80)	61% of patients had a minimum 10-point decrease in their ODI score (*p* < .01)	Non-reported

### Risk of bias

**Figure 2.**
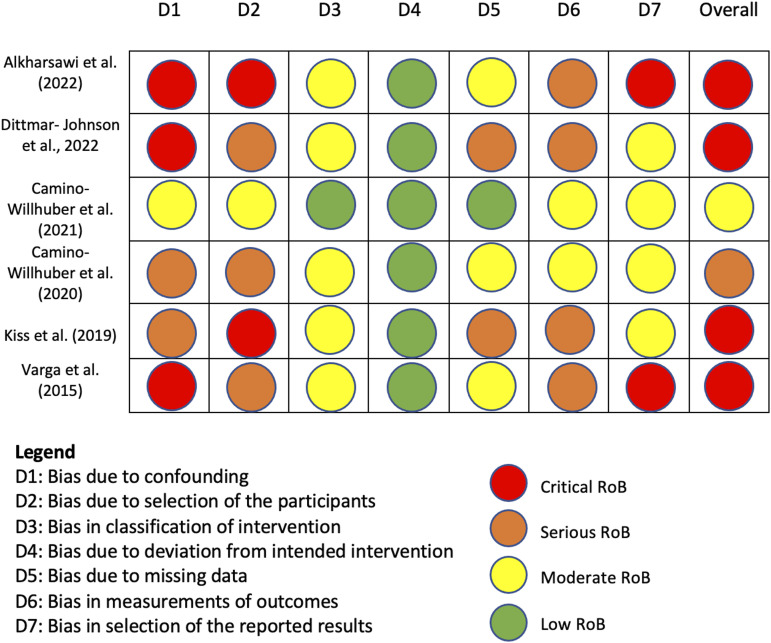
Risk of Bias in Non-Randomized Studies-I tool for assessment of the studies included in the systematic review.

The risk of bias (RoB) was assessed using the ROBINS-I tool. Critical RoB was found in 4/6 articles, serious RoB was found in 1/6 articles, and moderate RoB was found in 1/6 articles. Domains of most concern across studies were bias due to confounding, bias due to selection of participants, and bias in measurement of outcomes, see Figure 2.

#### Visual analog scale score

**Figure 3.**
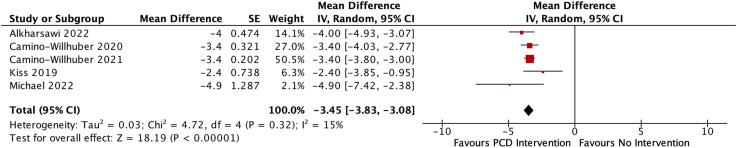
Forest plot showing the meta-analysis of Visual Analog Scale (VAS) scores for low back pain.

Among many tools used for pain assessment, the visual analog scale is a reliable tool to assess lower back pain severity. Of the full-text articles included in this review, 6/6 (100%) reported VAS scores. A within-group mean difference analysis was done, presented in Figure 3. Pooled analysis showed a significant difference between pre-PCD and post-PCD lower back pain scores (Weighted Mean Difference (WMD): -3.45; 95% CI: -3.83, -3.08; I^2^ = 15%; *p* < .001). Heterogeneity was low and not significant (I^2^ = 15%; Chi^2^ = 4.72, df = 4, *p* = .32). Data for Varga et al. were excluded from this analysis as neither the standard deviation, confidence interval, or standard error was reported for pre- and post-procedure VAS scores, but it should be noted that at 6 months of follow-up, there was a significant decrease in VAS score for low back pain, decreasing from 68 mm to 43 mm.

#### Oswestry disability index score

**Figure 4.**
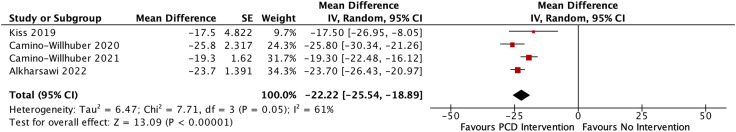
Forest plot showing the meta-analysis of Oswestry Disability Index (ODI) scores for functional disability.

Another common scoring system for patients with LBP is the Oswestry Disability Index. Among full-text articles included in this review, 5/6 (83%) of articles reported ODI scores. A within-group mean difference analysis was done, presented in Figure 4. Pooled analysis showed a significant difference between pre-PCD and post-PCD lower back pain scores (WMD: -22.22; 95% CI: -25.54, -18.89; *p* < .001). Heterogeneity was high and statistically significant (I^2^ = 61%; Chi^2^ = 7.71, df = 3, *p* = .05) Data for Varga et al. were excluded from this analysis as the post-procedure ODI scores were not reported, but it noted 61% of patients had a minimum 10-point decrease in their ODI score (*p* < .01).

#### Complications

Of the included studies, 4/6 reported post-PCD complications identified immediately after the procedure or at the follow-up time points (^7,12–14^). Complications identified in these studies encompassed various infections, such as skin infections (*n* = 5), urinary tract infections (*n* = 3), deep infections (*n* = 2), as well as superficial thrombophlebitis (*n* = 2). Some patients had to be readmitted within a 30-day period and required intravenous medication (*n* = 15). There were also documented instances of adjacent vertebral fractures (*n* = 2) and disc extrusion (*n* = 1). A few patients experienced foraminal decompression resulting from radicular symptoms (*n* = 2), cement leakage into the foramen (*n* = 2), decompression surgery (*n* = 5), and fusion surgery due to ongoing pain (*n* = 5). Some patients needed further operations because of persistent symptoms (*n* = 2), while others required additional PCD procedures at different levels due to recurring pain (*n* = 5).

#### Methodological quality and risk of bias

The Methodological Index for Non-Randomized Studies (MINORS) tool was used to assess the articles included in this study. Items 1–8 were used for the evaluation of the articles; each scored as either 0 (not reported), 1 (reported but inadequate), or 2 (reported and adequate). The median methodological quality of the articles was 9, with a range of 7–10. Areas of significant concern were items 3 (prospective collection of data) and 6 (follow-up period appropriate to the aim of the study).

## Discussion

Our systematic review and meta-analysis evaluated the effectiveness of PCD in managing degenerative disc diseases, specifically by examining the changes in lower back pain (using VAS scores) and functional disability (using ODI scores). Our analysis reveals a statistically and clinically significant improvement in both VAS and ODI scores following PCD.

PCD emerges as a viable option for managing low back pain resulting from advanced degenerative disc disease in elderly patients, as demonstrated by Varga et al.^4^ Comparing our results to previous research, we found similar evidence supporting the effectiveness of PCD in the management of degenerative disk diseases.^4–7^ Moreover, the efficacy of cement discoplasty is corroborated by studies such as that of Alkharsawi et al.,^13^ where cement injection into the affected disc space contributed to pain reduction and enhanced patient-reported outcomes. A retrospective study conducted by Camino et al.^7^ demonstrated the short-term effectiveness of PCD, with significant pain reduction as well as favorable radiological changes such as partial correction of lumbar lordosis and Cobb angle. In line with these findings, Kiss et al.^5^ also found that PCD not only enhances lumbar alignment but also leads to indirect foraminal decompression, ultimately contributing to pain relief and improved functional capacity. Collectively, these studies underscore PCD's potential as a valuable treatment option for elderly patients with degenerative disc disease, showcasing its capacity to alleviate pain, enhance stability, and improve overall functional outcomes through minimally invasive means.

Our study is the first meta-analysis on this topic, which is a significant contribution to the existing literature as it provides a more robust and comprehensive understanding of the evidence supporting this procedure as an effective treatment option for DDDs.

It is important consider post-PCD complications occurring immediately after the procedure or at follow-up time points. The most common complications were infections with readmission for IV medications.^7,12–14^ Overall, rates across multiple studies were low (<15%), offering a benefit compared to more invasive procedures.^5,7,12^ There remains a need for further investigation into the safety and long-term effectiveness of this treatment approach, as not all studies reported complications.

Despite our significant findings, there are several limitations to our study. Firstly, the small number of included articles, due to a shortage of published literature on this topic, may impact the generalizability of our results. Next, the included texts were all retrospective case-studies with no control groups, with variability between studies in the selection criteria of patients, reporting of complications, the underlying DDD, and other comorbidities that the patient(s) may have had. Follow-up times were another limitation, as the longest follow-up time in any study was 24 months. These factors along with others may have contributed to the significant heterogeneity observed in the ODI score, resulted in a low median MINOR score, and a high risk of bias. Therefore, it is important that these results be interpreted cautiously.

Considering these limitations, future research should focus on conducting high-quality randomized controlled trials with larger sample sizes and extended follow-up periods to further assess the effectiveness and safety of PCD in managing degenerative disc diseases, as well as any associated complications. Moreover, future studies should explore how patient characteristics, such as age, comorbidities, and disease severity, might impact the efficacy of PCD to better identify which patient subgroups could benefit the most from this treatment approach.

## Conclusion

Our systematic review and meta-analysis suggest that PCD may be an effective and safe intervention for managing degenerative disc diseases, as evidenced by clinically significant improvements in both pain and functional disability. However, it's important to acknowledge that the validity and generalizability of our findings are somewhat limited due to the methodological weaknesses and high risk of bias in the studies we included. Recognizing the limitations, there is a clear need for further research on this procedure in the management of DDDs. Our study offers preliminary insights into the potential benefits of PCD in managing degenerative disc diseases and serves as a foundation for guiding future research in this area.

## References

[1] Ravindra VM Senglaub SS Rattani A , et al. Degenerative lumbar spine disease: estimating global incidence and worldwide volume. Glob Spine J 2018; 8(8): 784–794. 10.1177/2192568218770769 PMC6293435 30560029

[2] Fernandez-Moure J Moore CA Kim K , et al. Novel therapeutic strategies for degenerative disc disease: Review of cell biology and intervertebral disc cell therapy. SAGE Open Med 2018; 6: 2050312118761674. 29568524 10.1177/2050312118761674 PMC5858682

[3] Wu PH Kim HS Jang IT . Intervertebral disc diseases part 2: a review of the current diagnostic and treatment strategies for intervertebral disc disease. Int J Mol Sci 2020; 21(6): 2135. 32244936 10.3390/ijms21062135 PMC7139690

[4] Varga PP Jakab G Bors IB , et al. Experiences with PMMA cement as a stand-alone intervertebral spacer: Percutaneous cement discoplasty in the case of vacuum phenomenon within lumbar intervertebral discs. Orthopä 2015; 44 Suppl 1(S1): 1–S7. 10.1007/s00132-014-3060-1 25875227

[5] Kiss L Varga PP Szoverfi Z , et al. Indirect foraminal decompression and improvement in the lumbar alignment after percutaneous cement discoplasty. Eur Spine J 2019; 28(6): 1441–1447. 31006068 10.1007/s00586-019-05966-7

[6] Fusini F Girardo M Aprato A , et al. Percutaneous cement discoplasty in degenerative spinal disease: systematic review of indications, clinical outcomes, and complications. World Neurosurg 2022; 168: 219–226. 36220492 10.1016/j.wneu.2022.10.008

[7] Camino Willhuber G Kido G Pereira Duarte M , et al. Percutaneous cement discoplasty for the treatment of advanced degenerative disc conditions: a case series analysis. Glob Spine J 2020; 10(6): 729–734. 10.1177/2192568219873885 PMC7383797 32707012

[8] Page MJ McKenzie JE Bossuyt PM , et al. The PRISMA 2020 statement: an updated guideline for reporting systematic reviews. BMJ 2021; 372: n71. 33782057 10.1136/bmj.n71 PMC8005924

[9] Slim K Nini E Forestier D , et al. Methodological index for non-randomized studies (minors): development and validation of a new instrument. ANZ J Surg. 2003 Sep; 73(9): 712–6. 12956787 10.1046/j.1445-2197.2003.02748.x

[10] Review manager (RevMan) [Computer program]. Version 5.4. Copenhagen: The Cochrane Collaboration, 2020.

[11] Sterne JA Hernán MA Reeves BC , et al. ROBINS-I: a tool for assessing risk of bias in non-randomised studies of interventions. BMJ 2016; 355: i4919. 27733354 10.1136/bmj.i4919 PMC5062054

[12] Camino-Willhuber G Norotte G Bronsard N , et al. Percutaneous cement discoplasty for degenerative low back pain with vacuum phenomenon: a multicentric study with a minimum of 2 years of follow-up. World Neurosurg 2021; 155: e210–e217. 34403794 10.1016/j.wneu.2021.08.042

[13] Alkharsawi M Shousha M Boehm H , et al. Cement discoplasty for managing lumbar spine pseudarthrosis in elderly patients: a less invasive alternative approach for failed posterior lumbar spine interbody fusion. Eur Spine J 2022; 31(7): 1728–1735. 35347424 10.1007/s00586-022-07186-y

[14] Michael DJH Francisco CL Eduardo GC , et al. Percutaneous cement discoplasty in the treatment of degenerative disc disease. CASE SERIES. Coluna/Columna 2022; 21(1): e259477.

